# Novel Synthesis of Holey Reduced Graphene Oxide/Polystyrene (HRGO/PS) Nanocomposites by Microwave Irradiation as Anodes for High-Temperature Lithium-Ion Batteries

**DOI:** 10.3390/ma12142248

**Published:** 2019-07-12

**Authors:** Yazeed Aldawsari, Yasmin Mussa, Faheem Ahmed, Muhammad Arsalan, Edreese Alsharaeh

**Affiliations:** 1 College of Science and General Studies, Alfaisal University, P.O. Box 50927, Riyadh 11533, Saudi Arabia; 2 EXPEC Advanced Research Center, Saudi Aramco, P.O. Box 5000, Dhahran 31311, Saudi Arabia

**Keywords:** Li-ion batteries, holey reduced graphene oxide, polymer, thermal management, high temperature

## Abstract

To overcome the risk of exothermic lithium-ion battery overheating reactions, we fabricated a novel, high-temperature-stable anode material composed of holey reduced graphene oxide/polystyrene (HRGO/PS) nanocomposites synthesized through in situ bulk polymerization in the presence of HRGO via microwave irradiation. The HRGO/PS nanocomposites were characterized by Fourier transform infrared spectroscopy, X-ray diffraction, Raman spectroscopy, and electron microscopy analyses including field-emission scanning electron microscopy and transmission electron microscopy. All characterization studies demonstrated homogenous dispersion of HRGO in the PS matrix, which enhanced the thermal and electrical properties of the overall nanocomposites. These novel HRGO/PS nanocomposites exhibited excellent electrochemical responses, with reversible charge/discharge capacities of 92.1/92.78 mA·h/g at a current density of 500 mA/g with ~100% capacity retention and ~100% coulombic efficiency at room temperature. Furthermore, an examination of the electrochemical properties of these nanocomposites at 110 °C showed that HRGO/PS nanocomposites still displayed good charge/discharge capacities with stable cycle performances for 150 cycles.

## 1. Introduction

Lithium-ion (Li-ion) batteries are rechargeable batteries based on the intercalation and deintercalation of lithium ions. Li-ion batteries were first commercialized by Sony in 1991 [1]. These batteries are found in various applications as an energy storage unit with a huge market share in electric vehicles and electronic devices [2]. Li-ion batteries are commercially available because of their high capacity, long life, and environmental friendliness. The main components of a Li-ion battery are the electrodes (cathode and anode), binder for the electrodes, separator, and electrolyte. These components play a major role in the overall performance of lithium-ion batteries.

Lithium metal is the choice for the anode in a Li-ion battery because of its highest theoretical capacity (3860 mA·h/g) in comparison to other potential anode material candidates. However, challenges arise in using lithium as anode material since lithium ions have a tendency to form or deposit in dendritic forms. It is the formation of dendritic lithium that results in thermal runaway. When considering the safety of Li-ion batteries, dendrite-free lithium needs to be obtained while, at the same time, good electrochemical performance is maintained [3].

Recently, polymeric electrodes have been used in Li-ion batteries as an alternative material because of their high mechanical strength and environmental friendliness [4]. Conductive polymers have been primarily considered because of their good affinity to other materials, electrical conductivity that reaches 10^3^ S/cm, and ease of preparation via various polymerization methods [5]. The most commonly used conductive polymers in Li-ion batteries include polythiophene (PT), polypyrrole (PPy), polyaniline (PANI), poly(3,4-propylenedioxythiophene) (PProDOT), poly(3,4-ethylenedioxythiophene) (PEDOT), and PEDOT:poly(4-styrene sulfonate) (PEDOT:PSS) [6].

In addition to conductive polymers, neutral conjugated polymers with lower conductivity in the range of 1010–105 S/cm are also known. Doping and other modification processes can enhance the electrical conductivity of the materials and make them act as conductive polymers. This approach can be followed to enhance the electrical properties of insulating polymers or nonconjugated polymers such as polypropylene, polyethylene, poly(ethylene terephthalate), or polystyrene [7].

Nonconjugated polymers including polystyrene showed an enhancement in the electrical properties after the addition of graphene, making them promising components of rechargeable Li-ion batteries [8,9].

Not many studies are reported for graphene-based polymer composites as anode materials in lithium-ion batteries. However, Song et al. reported the use of graphene-based polymer nanocomposites consisting of graphene and two polymer materials, namely, poly(anthraquinonyl sulfide) and polyimide as cathode materials. The polymer–graphene nanocomposites were prepared by an in situ polymerization method. The highly dispersed graphene in the polymer-based nanocomposite material resulted in fast charging and discharging, obtaining more than 100 mA·h/g within only a few seconds [10].

Although this abovementioned example demonstrates the potential application of graphene-based polymer composites as high-performance electrodes in Li-ion battery applications, there is a need to investigate them to meet higher energy demands and their suitability for safe use in a wide range of operating conditions, especially harsh environments. Some of the fields that are demanding robust rechargeable energy storage systems include the oil industry, aerospace, and hybrid car markets. Energy storage systems used in the oil field industries must be able to withstand a variety of operating temperatures (up to temperatures of 150 °C and higher). High-temperature electronics are also used in the aviation industry, especially for those systems operating near the engine. The aerospace industry also needs high-temperature energy storage systems to power devices exploring planet surfaces. The engine, transmission, and brake systems of hybrid vehicles require electronics to operate under elevated temperatures ranging from 200 to 800 °C [11,12].

Graphene-based polymer composites are good candidates for thermal management applications because of the superior thermal conductivity of graphene [9,13,14,15], which enables the development of electrode materials for Li-ion batteries operating at high temperatures [16]. However, there is limited knowledge on graphene-based polymer nanocomposites as electrode materials for rechargeable Li-ion batteries at high operating temperatures.

Recently, we have reported HRGO as an anodic material that shows good performance in Li-ion rechargeable batteries [17]. In this study, we report in situ bulk synthesis of HRGO/PS nanocomposites with low (0.1%, 1%, and 2%) HRGO loading via in situ bulk polymerization using microwave irradiation. These nanocomposites are further investigated as anodic materials for Li-ion batteries at high temperatures.

## 2. Experimental Details

### 2.1. Materials

Styrene (S) monomer (99%) purchased from Sigma_ Aldrich. (St. Louis, MO, USA) was stored in a refrigerator and used as received. Benzoyl peroxide (BP) was obtained from BDH Chemicals Ltd. (Dubai, UAE) and was used as an initiator.

### 2.2. Preparation of Holey Reduced Graphene Oxide/Polystyrene (HRGO/PS) Nanocomposites

The HRGO was first synthesized by deposition of silver (Ag) nanoparticles onto reduced graphene oxide (RGO) sheets, followed by nitric acid treatment to remove Ag nanoparticles by microwave irradiation to form a porous structure. The detailed synthetic procedure of HRGO is described in our previous work [17]. A mixture of styrene (S, 2.0 g), different wt.% (0.1%, 1%, and 2%) of HRGO, and benzoyl peroxide (BP, 0.1 g) initiator was sonicated for 1 h, and then the mixture was maintained at 60 °C for 20 h to promote in situ free radical bulk polymerization. Next, this mixture was sonicated for 1 h, followed by reduction using microwave irradiation. The neat PS was prepared for comparison using the same procedure but without the addition of HRGO.

### 2.3. Material Characterization

Fourier transform infrared (FTIR) spectra of the nanocomposites were acquired using a Thermo Scientific Nicolet-iS10 FTIR and were recorded in the range of 4000–400 cm^−1^. X-ray diffraction (Philips-PW 1729, Eidenhoven, The Netherlands) was used to study the phase composition and crystalline nature of the nanocomposites with Cu radiation [30 kV, 40 mA, Kα radiation (1.54430 Å)]. Scanning electron microscopy (SEM) images were obtained using an FEI Quanta 200 SEM (Hillsboro, OR, USA) after they were mounted on the nanocomposite slabs and coated with gold via a Bio-Rad Polaron E6100 sputtering system (FEI, Hillsboro, OR, USA). High-resolution transmission electron microscopy (HRTEM) images were obtained using a JEOL JSM-2100F HRTEM (JOEL, Tokyo, Japan) operated at 200 kV. A drop of the specimen dispersed in ethanol was placed on copper grids and dried for the studies. Raman spectra were recorded using a Bruker Equinox 55 FTIR spectrometer (Bruker, Berlin, Germany) equipped with an FRA106/S FT-Raman module (Bruker, Berlin, Germany) and a liquid N_2_-cooled Ge detector (Bruker, Berlin, Germany) using the 1064 nm line of a Nd:YAG laser with an output laser power of 200 mW. Thermogravimetric analyses (TGA) were performed using a HITACHI STA 7200 thermogravimetric analyzer (HITACHITokyo Japan) under a nitrogen atmosphere at a heating rate of 10 °C/min. Differential scanning calorimetry (DSC) studies were performed using a HITACHI DSC 7020 instrument (HITACHI, Tokyo, Japan). A required amount (2–5 mg) of electrode material was used and crimp-sealed in an aluminum sample holder. DSC measurements were performed under a nitrogen atmosphere at a heating rate of 2 °C/min.

### 2.4. Electrical Characterization

Resistance of the nanocomposites was calculated using the two-probe method in a two-electrode (Cu) configuration using a Keithley 4200 SCS-four-probe electrical current-voltage (I–V) measurement system (Kethly, Roma, Italy). Conductivities of the nanocomposites were calculated by fitting their I–V characteristics for 10 cycles. After the measurements were performed, the mass of each film containing the nanocomposite was measured, and the effective thickness of each film was calculated to obtain the film conductivity from the measured resistance.

### 2.5. Electrochemical Characterization

The electrode was fabricated by using 60% of the active material in addition to 30% Super P conductive carbon and 10% Polyvinylidene Flourid (PVDF) binder [10] in N-Methyl-2-pyrrolidone (NMP) solvent, which was then cast on a copper foil with a thickness of 100 µm. The electrode was then dried for several hours at 80 °C. The electrode was cut and had a diameter of 15 mm. The mass of the active material (including the conductive agent and binder) on the current collector was around 1 mg. The specific capacity was obtained by dividing the capacity by the weight of the active material. The electrochemical performance was evaluated using a two-electrode coin half-cell with a Celgard 2325 polypropylene membrane as the separator and lithium metal (purity 99.9%) as the counter electrode; 1 M LiPF_6_ in a mixture of ethylene carbonate and dimethyl carbonate (EC/DMC) (1:1, v/v) was used as the electrolyte. Prior to assembly, the electrode was pre-lithiated by direct contact with lithium foil for 20 min. For comparison purposes, a non-pre-lithiated electrode was also used. The cells were then assembled in a dry room and were galvanostatically charged/discharged using a Gamry potentiostat. Cyclic voltammetry (CV) analyses were performed using a Gamry 3000 electrochemical working station at a scan rate of 30 mV/s in a voltage range of 0–3 V. Electrochemical impedance spectroscopy (EIS) was also performed using a Gamry 3000 electrochemical working station by applying a perturbation voltage of 10 mV for frequencies between 1 Hz and 100 kHz at room temperature and open circuit voltage (OCV). Moreover, high-temperature tests were conducted through a bomb calorimeter vessel by connecting the positive and negative terminal of the battery to the two electrodes of the vessel. The battery was kept inside the vessel at 110 °C overnight to ensure thermal equilibrium was reached. Electrochemical measurements, which included galvanostatic charge/discharge, CV, and EIS, were then obtained by connecting the bomb calorimeter vessel to the Gamry potentiostat.

## 3. Results and Discussion

**Figure 1:**
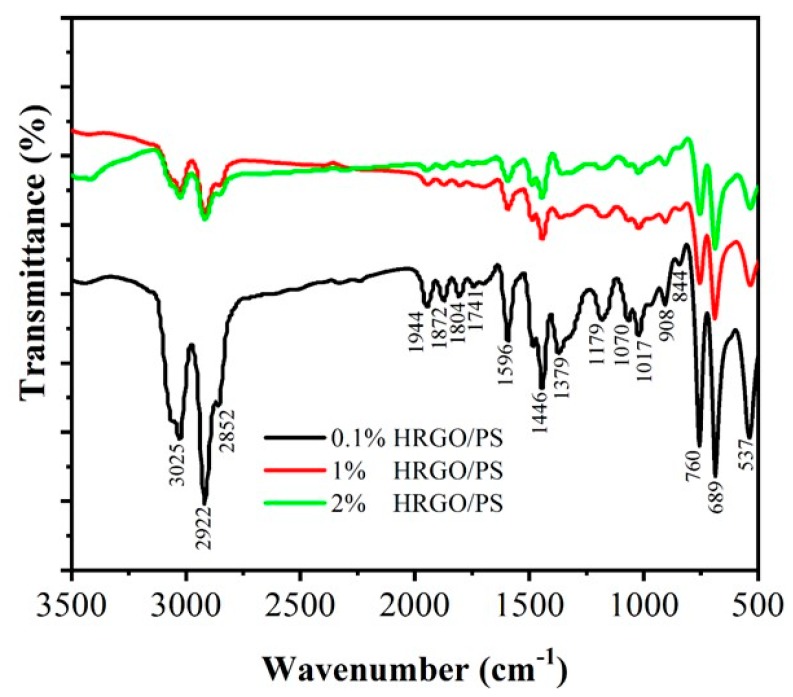
Fourier transform infrared (FTIR) spectra of 0.1% holey reduced graphene oxide/polystyrene (HRGO/PS), 1% HRGO/PS, and 2% HRGO/PS nanocomposites.

FTIR spectral analysis was performed to confirm the chemical structure of the PS on the HRGO sheet. Figure 1 shows the FTIR spectra of 0.1% HRGO/PS, 1% HRGO/PS, and 2% HRGO/PS nanocomposites. The peaks at approximately 2800–3060, 2800–3000, 1550–1750, 1550–1610, 1300–1380, 880–1000, and 625–970 cm^−1^ correspond to aliphatic C–H stretching, aromatic C–H stretching, C=O, C=C stretching, CH_2_ bending, C–O stretching, and the characteristic sensitive vibration modes of polystyrene as shown in Figure S1, respectively. While the peak intensities changed when PS formed composites with HRGO, there was no shift in the peak positions for HRGO/PS nanocomposites when compared to pure PS, indicating no change in the inner structure of PS [18].

**Figure 2:**
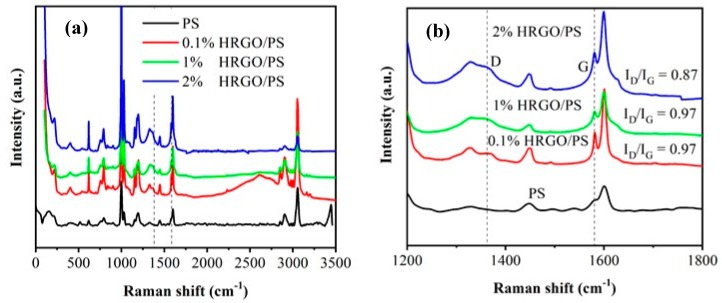
(**a**) and (**b**) Raman spectra of 0.1% HRGO/PS, 1% HRGO/PS, and 2% HRGO/PS nanocomposites indicating the D and G bands.

Figure 2a,b shows the Raman spectra of PS and HRGO/PS nanocomposites. Sufficient information about the structure of graphene can be extracted from the Raman spectrum. In addition to the PS modes, the peaks at 1358 and 1580 cm^−1^ were observed and are attributed to the sp^3^ (D band) and sp^2^ (G band) hybridized carbon atoms, respectively, for the HRGO/PS nanocomposites. Comparing with PS, the D-band from HRGO almost overlapped with the PS band in HRGO/PS, and the intensities of the characteristic modes of HRGO and PS were found to be modified by increasing the HRGO% in the PS matrix. The intensities of D/G ratios of 0.1%, 1%, and 2% HRGO in PS were found to be 0.97, 0.97, and 0.87, respectively. A higher intensity ratio of D/G was shown for 0.1% and 1%, which means a smaller size of sp^2^ domains [19].

**Figure 3:**
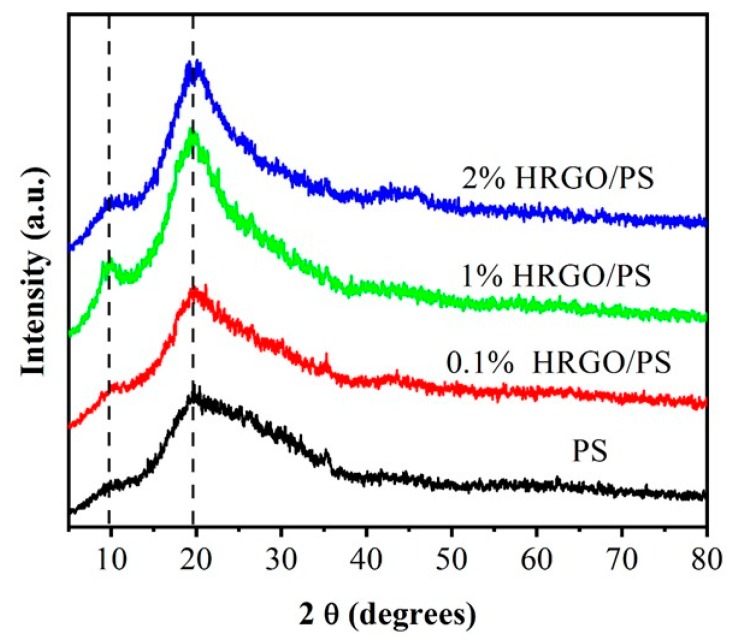
XRD patterns of 0.1% HRGO/PS, 1% HRGO/PS, and 2% HRGO/PS nanocomposites.

Figure 3 shows the XRD patterns of the PS and HRGO/PS nanocomposites. The XRD pattern of PS exhibited two main broad diffraction peaks at 9.38° that were the polymerization peaks due to the intermolecular backbone–backbone correlation. The size of the side group, which matched the hexagonal ordering of the molecular chains, and the other amorphous halo peak at 19.7° were attributed to the van der Waals distance. HRGO/PS (0.1%) showed similar XRD peaks to PS, while the XRD peaks of 1% and 2% HRGO/PS became narrower and more intense, indicating some kind of crystalline structure formation [20]. Mainly, it could be attributed to a relatively intense number of staked graphene layers with the increase in HRGO content. Moreover, no change in peak position was observed with the increase in HRGO content.

**Figure 4:**
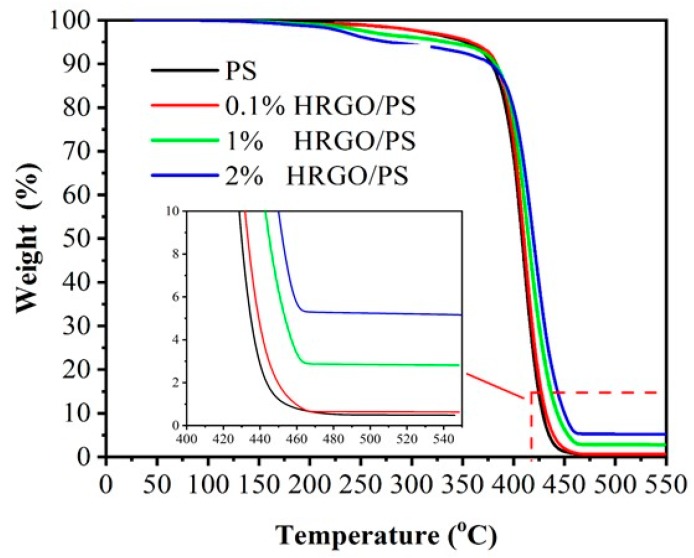
Thermogravimetric analysis (TGA) curves of 0.1% HRGO/PS, 1% HRGO/PS, and 2% HRGO/PS nanocomposites.

**Figure 5:**
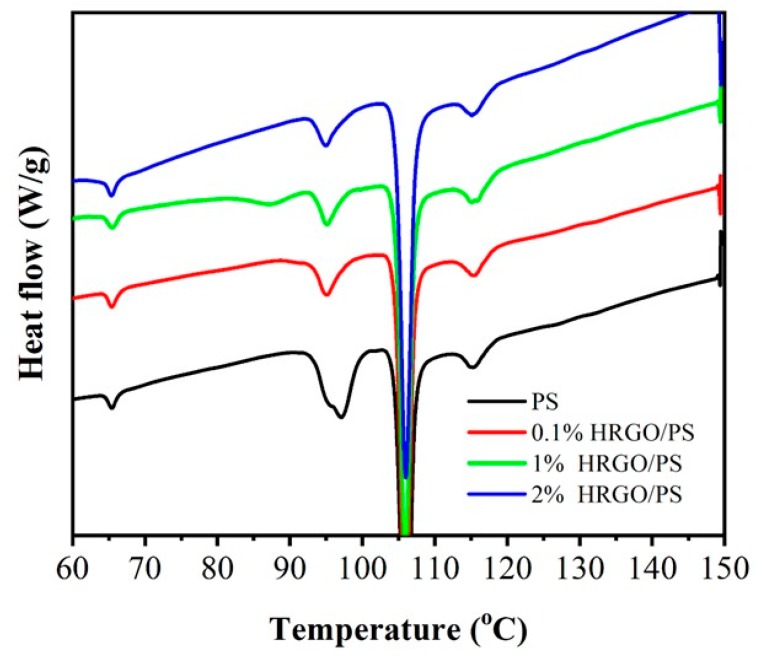
Differential scanning calorimetry (DSC) curves of 0.1% HRGO/PS, 1% HRGO/PS, and 2% HRGO/PS nanocomposites.

To determine the effects of HRGO on the thermal stability of the nanocomposites, the thermal properties of PS and the HRGO/PS nanocomposites with various concentrations of HRGO ranging from 0.1% to 2% were investigated by TGA and DSC; the obtained TGA plots of PS and the nanocomposites are shown in Figure 4. It was observed that the decomposition of pristine PS started at ~350 °C, and PS completely degraded at 430 °C, which was because of the main-chain pyrolysis [21]. In comparison to PS, 1% and 2% HRGO/PS composites exhibited better thermal stability, which was due to the interaction between HRGO and PS, while the 0.1% composite decomposed easily in high temperature. The highest thermal stability was found in the case of 1% HRGO/PS, which had a 3% total weight loss. Figure 5 shows the DSC plots of PS and HRGO/PS nanocomposites. For the pristine PS matrix, Tg was observed at 93.9 °C, and the presence of HRGO showed no significant Tg changes in the resulting PS/HRGO nanocomposites. This could be due to the bonding between the HRGO and PS, which was not that strong, because of the limited existence of oxidized functional groups or reactive C=C on the HRGO surface for the monomer to chemically react with during polymerization [22]. However, 1% HRGO/PS could have stronger interfacial interactions between the HRGO and PS matrix caused by the presence of short to moderate PS chains bonded to HRGO, which was clear from the Tg peak at 85 °C that appeared only in 1% HRGO/PS, thus improving the properties of the 1% HRGO/PS nanocomposites [23].

**Figure 6:**
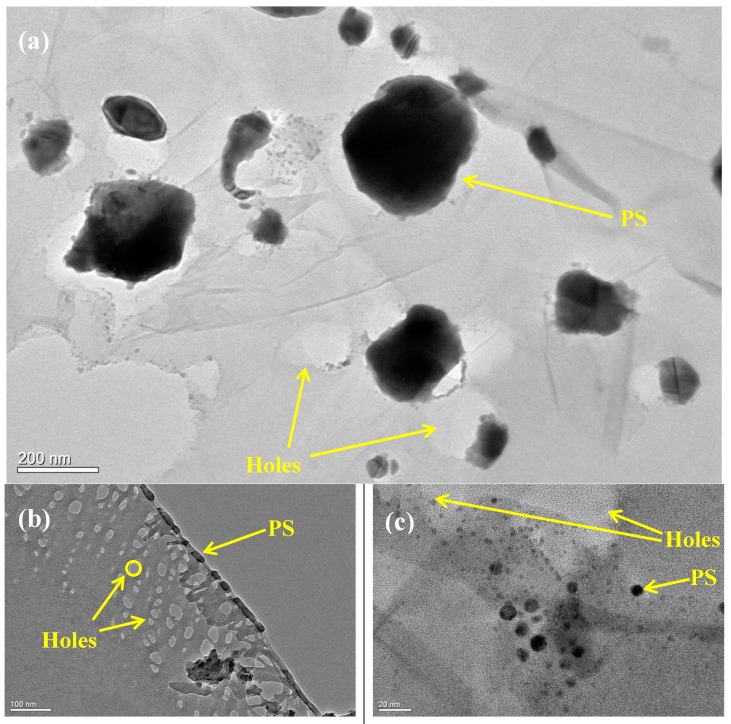
HR-TEM images of the HRGO/PS nanocomposites at (**a**) 200, (**b**) 100, and (**c**) 20 nm.

The morphology of the as-prepared samples was characterized using high-resolution transmission electron microscopy (HR-TEM). The HR-TEM images of the HRGO/PS nanocomposites are shown in Figure 6 under different magnifications, which show the dispersion of HRGO within the PS matrix.

**Figure 7:**
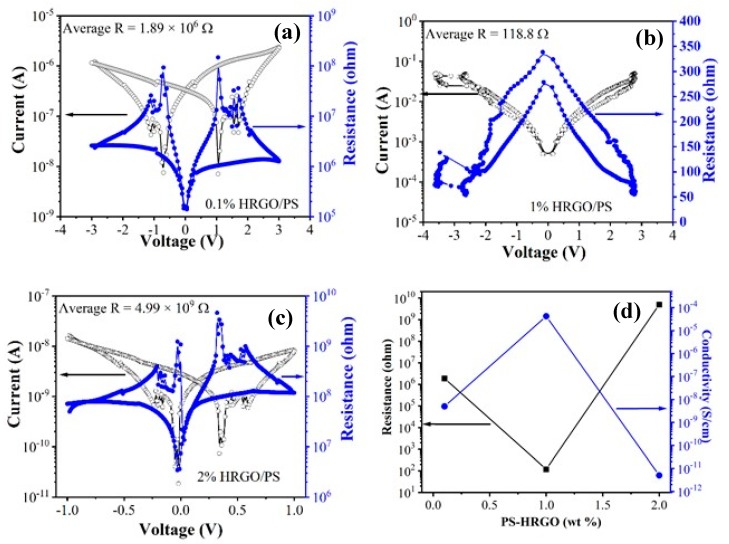
I–V characteristics of HRGO/PS nanocomposites containing (**a**) 0.1% HRGO/PS, (**b**) 1% HRGO/PS, and (**c**) 2% HRGO/PS. (**d**) Plot of electrical conductivity as a function of HRGO concentration in the HRGO/PS nanocomposites.

Figure 7 and Table 1 show the electrical conductivity results for the HRGO/PS nanocomposites. Neat PS is insulating and showed electrical conductivity values in the range of 10^−2^–10^−7^ S/cm. The plotted data display a power law dependence on the HRGO concentration. This result indicates that the electrical conductivity of the nanocomposites follows the percolation model. The electrical conductivity of the HRGO/PS nanocomposites increased as the HRGO concentration increased from 0.1% to 1% and then decreased for a further HRGO concentration increase to 2%. For 1% HRGO/PS, the conductivity increased by 2 orders of magnitude (4.47 × 10^−5^ S/cm) compared to that of 0.1% HRGO/PS (4.98 × 10^−9^ S/cm), while it was ~3 times higher than that of the 2% HRGO/PS nanocomposites (4.88 × 10^−12^ S/cm). This conductivity enhancement in 1% HRGO/PS nanocomposites may be due to the large surface area of the HRGO sheets (457 m^2^/g) [17], where such high surface area materials act as effective percolative conducting bridges. At slightly higher concentrations of HRGO, the increase in the conductivity may be due to the interaction between the HRGO layers and PS for which electron mobility inside the composite system increases. It can be explained that the electrical conductivity of the composites increases significantly once the conductive networks of HRGO have formed above a certain critical concentration of the fillers in the matrix. Moreover, despite the increased content of HRGO in 2% HRGO/PS nanocomposites, the decrease in conductivity noted could be due to the insufficient dispersion of HRGO throughout the polystyrene matrix. Similar problems were also reported by other researchers [24]. On the other hand, the obtained electrical conductivities were slightly lower than the results reported in the literature [8].

Other factors for the enhanced electrical properties in 1% HRGO/PS included the well dispersity of 1% HRGO/PS, which was confirmed by Raman as 1% HRGO/PS showed larger sp^3^ domains when compared to 2% HRGO/PS. Similarly, XRD of 1% HRGO/PS indicated a higher degree of crystallization when compared to 2% HRGO/PS, indicating a higher degree of crystallization. Moreover, the appearance of an extra Tg in 1% HRGO/PS is an indication of the strong interaction of 1% HRGO/PS with short to moderate PS chains. All could be possible factors in enhancing the properties of 1% HRGO/PS.

**Figure 8:**
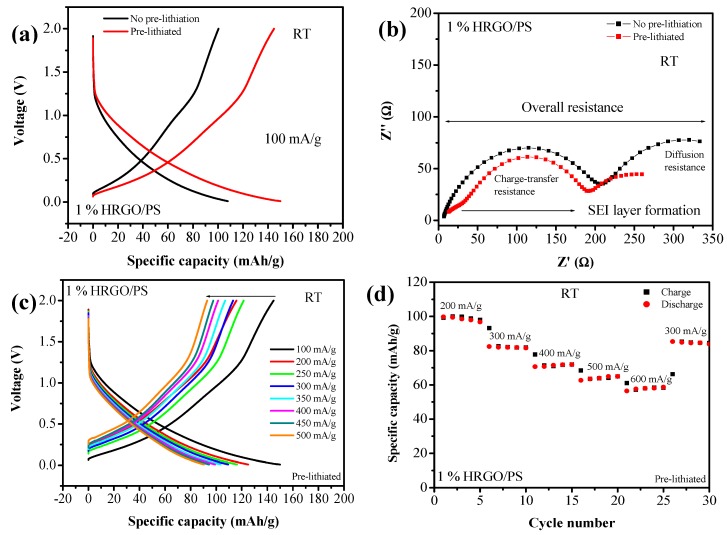
(**a**) Galvanostatic charge/discharge curves for non-pre-lithiated and pre-lithiated 1% HRGO/PS electrode for one cycle obtained at a current density of 100 mA/g, (**b**) Nyquist plot for non-pre-lithiated and pre-lithiated 1% HRGO/PS electrodes, and (**c**) and (**d**) rate capabilities of pre-lithiated 1% HRGO/PS at various current densities.

To evaluate the electrochemical performance of the 1% HRGO/PS nanocomposites, we investigated the electrochemical response in terms of Li intercalation and deintercalation for one cycle as shown in Figure 8a. Figure 8a shows the galvanostatic charge/discharge curves of both non-pre-lithiated and pre-lithiated HRGO/PS electrodes from 0.01 to 2.0 V at a current density of 100 mA/g. The pre-lithiated electrode had a higher specific charge and discharge capacity of 144.88 and 149.97 mA·h/g, respectively, whereas the non-pre-lithiated electrodes exhibited charge and discharge specific capacities of 100.45 and 107.85 mA·h/g, respectively. An increase of ~40 mA·h/g was observed, which was due to the pre-treatment of the electrode, as pre-lithiation can prevent active lithium loss and thus obtain the maximal capacity [25].

Figure 8b shows the electrochemical impedance spectra (EIS) in the form of a Nyquist plot for both pre-lithiated and non-pre-lithiated 1% HRGO/PS nanocomposites. The Nyquist plot is composed of a semicircle in the mid-frequency region and a linear tail in the low-frequency regions, which are ascribed to the charge transfer and mass transfer of Li^+^ ions, respectively. The diameter of the semicircle of pre-lithiated 1% HRGO/PS was slightly smaller than the non-pre-lithiated 1% HRGO/PS. Interestingly, the pre-lithiated 1% HRGO/PS exhibited a semicircle in the high-frequency region, which was due to the formation of a well-defined solid electrolyte interphase (SEI) at the negative electrode without the need for formation cycles [26].

To further evaluate the high performance of the pre-lithiated 1% HRGO/PS, the rate capability was also investigated. Figure 8c shows the charge/discharge voltage profiles of the 1% HRGO/PS electrode at various current densities ranging from 100 to 500 mA/g. As the current density increased, the discharge potential decreased and the charge potential increased, which was due to kinetic effects of the material [27]. It was observed that the discharge capacity was about 149.97 mA·h/g at a low-current density of 100 mA/g, and then it reduced to 125.08, 109.4, and 99.15 mA·h/g at current densities of 200, 300, and 400 mA/g, respectively. Even at a high-current density of 500 mA/g, the electrode can deliver a discharge capacity of 90.38 mA/g with a reversible capacity of 100% as shown in Figure 8d.

**Figure 9:**
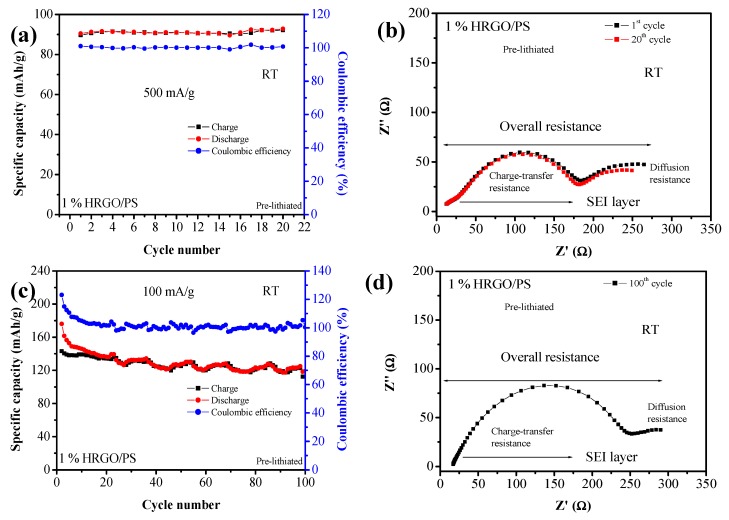
(**a**) Cycling performance of pre-lithiated 1% HRGO/PS at a current density of 500 mA/g. (**b**) Nyquist plot of pre-lithiated 1% HRGO/PS electrodes after the 1st and 20th cycle. (**c**) Cycling performance of pre-lithiated 1% HRGO/PS at a current density of 100 mA/g for 100 cycles. (**d**) Nyquist plot of pre-lithiated 1% HRGO/PS electrodes after the 100th cycle.

The cyclic performance test for the pre-lithiated electrodes were also quite remarkable, as shown in Figure 9a. At a current density of 500 mA/g, specific charge and discharge capacities of 92.1 and 92.78 mA·h/g were retained for 20 cycles, with coulombic efficiency and capacity retention of ~100% after the 20th cycle. The superior transport kinetics of the pre-lithiated 1% HRGO/PS were further confirmed by electrochemical impedance. Figure 9b shows the Nyquist plots after the 1st and 20th cycles. After 20 cycles at 500 mA/g, the charge transfer resistance of the electrode did not increase, confirming the highly reversible lithium storage capability of the 1% HRGO/PS with a stable SEI layer. Furthermore, the cycling performance of pre-lithiated 1% HRGO/PS at a current density of 100 mA/g for 100 cycles was also performed (Figure 9c). An initial discharge capacity of 176.17 mA·h/g was obtained with a reversible discharge capacity of 119.03 mA·h/g after 100 cycles. For the Nyquist plots, after 100 cycles a slight increase in the charge transfer resistance was observed (Figure 9d).

**Figure 10:**
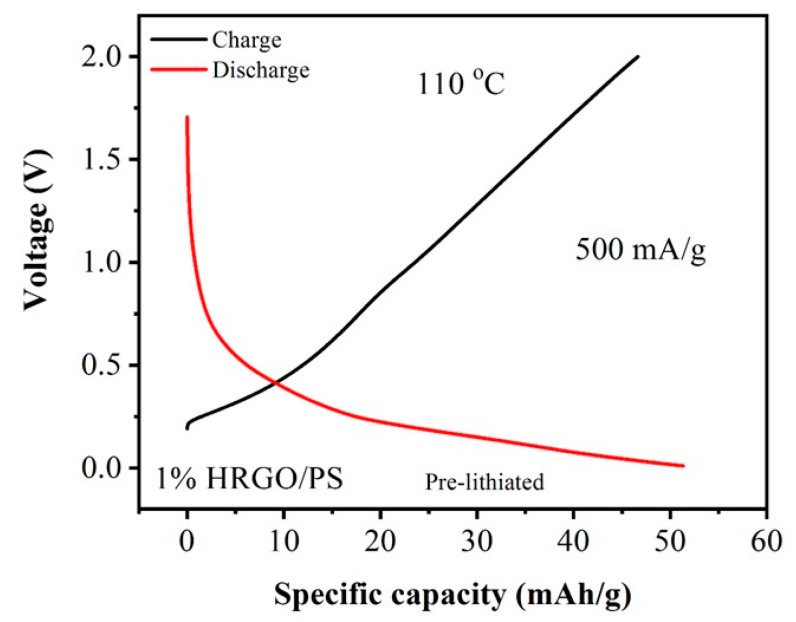
Galvanostatic charge/discharge curves for 1% HRGO/PS nanocomposites obtained at a current density of 500 mA/g at 110 °C.

To study the electrochemical performance of 1% HRGO/PS at 110 °C, galvanostatic charge/discharge cycling was performed for one cycle with a current density of 500 mA/g in a voltage window of 0.01–2.0 V, as shown in Figure 10. The initial cycling charge and discharge resulted in a specific capacity of 46.63 and 51.33 mA·h/g, respectively. Comparing the discharge specific capacities obtained at room temperature (90.38 mA/g) and 110 °C (51.33 mA·h/g) at the same current density (500 mA/g), a ~40% reduction in capacity was observed, which was due to the thermal decomposition of the SEI layer at elevated temperatures (>110 °C) [28].

**Figure 11:**
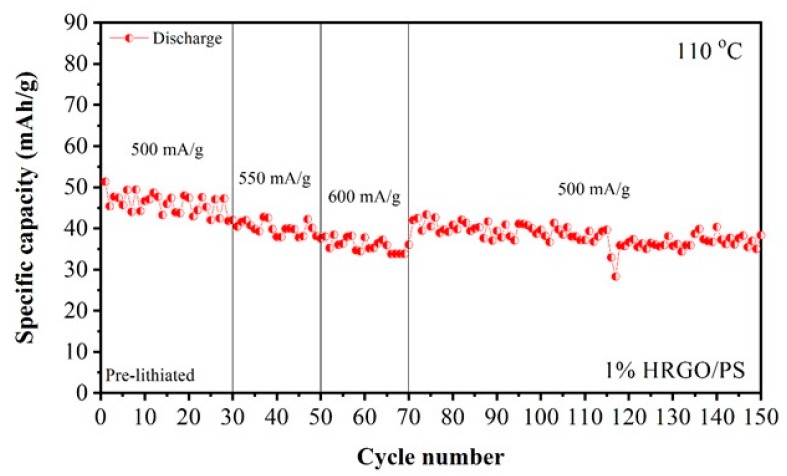
Rate capability at various current densities and cycling performance of 1% HRGO/PS at 110 °C.

Although the SEI layer degraded, the 1% HRGO/PS electrode material was still stable and had good cyclability at elevated temperatures, as shown from the rate capability and cycling performance in Figure 11. HRGO/PS (1%) exhibited excellent rate capability at various discharge densities (500, 550, and 600 mA/g), and the results are shown in Figure 11. After cycling at higher current densities, the cell was subjected to cycling again at a lower current density to evaluate the stability of the 1% HRGO/PS for varying current densities at elevated temperatures. The discharge capacity of the 1% HRGO/PS electrode at different current densities was 42 mA·h/g (500 mA/g) at the 30th cycle, 37.57 mA·h/g (550 mA/g) at the 50th cycle, 36.07 mA·h/g (600 mA/g) at the 70th cycle, and 38.35 mA·h/g (500 mA/g) at the 150th cycle, evidencing a good rate capability of 1% HRGO/PS with stable cycling performance for 150 cycles at 110 °C.

**Figure 12:**
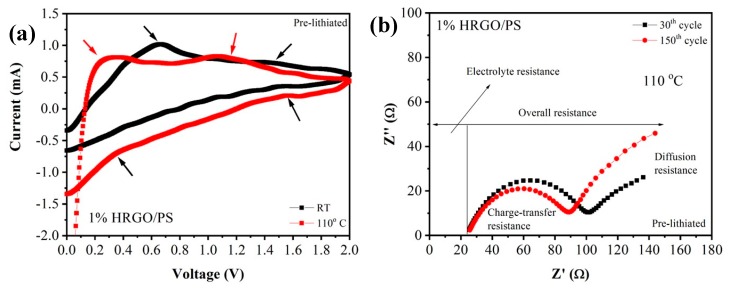
(**a**) Cyclic voltammetry (CV) of pre-lithiated 1% HRGO/PS electrodes and (**b**) Nyquist plot of pre-lithiated 1% HRGO/PS electrodes after the 30th and 150th cycle at 110 °C.

Figure 12a shows the CV curves of the prepared 1% HRGO/PS at both room temperature and 110 °C. During the cathodic (discharge) cycle, the segment between 1.5 and 0.4 V may correspond to lithium intercalation into HRGO/PS. In the anodic (charge) cycle, the voltage plateaus at 0.2 and 1.1 V corresponded to the anodic peaks of lithium deintercalation, which shifted to lower voltages at 110 °C. The redox peaks were weak, which could be due to the low content of HRGO [29]. Because of the weak redox peaks, it was difficult to obtain information related to the SEI layer. As expected, the Nyquist plots in Figure 12b further confirmed the decomposition of the SEI layer, increasing the resistance of the electrolyte. The increased resistance of the electrolyte was also affected by temperature. Interestingly, the charge-transfer resistance of the 1% HRGO/PS decreased when performed at 110 °C after 150 cycles. This enhanced performance at a high temperature could be explained through an Arrhenius equation, that is, the rate of the reaction increases with temperature. Also, the electrical/ionic conductivity of the electrolyte increased with increasing temperature because there was a drop in the ohmic potential or a decrease in the internal resistance of the cell with increasing temperature [30].

## 4. Conclusions

We have successfully incorporated HRGO within a polystyrene matrix via in situ bulk polymerization using microwave irradiation. FTIR, XRD, and Raman analyses as well as morphological characterization confirmed that the obtained nanocomposites exhibited a porous morphology and there was good dispersion of HRGO in the PS matrix, with 1% HRGO/PS exhibiting the highest electrical conductivity (4.47 × 10^−5^ S/cm). Electrochemical performances of the nanocomposites showed a reversible charge/discharge capacity of 92.1/92.78 mA·h/g at a high-current density of 500 mA/g with coulombic efficiency and capacity retention of ~100% at room temperature. In addition, electrochemical studies of these nanocomposites at a high temperature (110 °C) show that the 1% HRGO/PS nanocomposites displayed good charge and discharge capacities with stable cycle performances for 150 cycles at 110 °C. No short circuits, thermal runaway events, or capacity decay were observed. This study will open up a new direction for the potential applications of HRGO as a filler for a polymer matrix, with their conductive nature and their porous structure enabling their use as advanced electrode materials in high-temperature-stable energy storage applications.

## Figures and Tables

**Table 1:** Summary of the electrical properties of PS and HRGO/PS nanocomposites.

Sample	Electrical Conductivity (S/cm)
PS	10^−2^–10^−7^
0.1% HRGO/PS	4.98 × 10^−9^
1% HRGO/PS	4.47 × 10^−5^
2% HRGO/PS	4.88 × 10^−12^

## Supplementary Materials

The following are available online at https://www.mdpi.com/1996-1944/12/14/2248/s1, Figure S1: FTIR spectra of pure polystyrene.
