# Supplementary material for: Novel Synthesis of Holey Reduced Graphene Oxide/Polystyrene (HRGO/PS) Nanocomposites by Microwave Irradiation as Anodes for High-Temperature Lithium-Ion Batteries
Source: Materials (Basel). 2019 Jul 12;12(14):2248. doi: 10.3390/ma12142248 (PMC6678407; doi:10.3390/ma12142248)
Supplement: Supplementary file 1 [file materials-12-02248-s001.pdf]

*materials*

*Supplementary Information*

# Novel Synthesis of Holey Reduced Graphene Oxide/Polystyrene (HRGO/PS) Nanocomposites by Microwave Irradiation as Anodes for High-Temperature Lithium-Ion Batteries

**Yazeed Aldawsari [1], Yasmin Mussa [1], Faheem Ahmed [1], Muhammad Arsalan [2] and Edreese Alsharaeh [1,*]**

[1] College of Science and General Studies, Alfaisal University, P.O. Box 50927, Riyadh 11533, Saudi Arabia
[2] EXPEC Advanced Research Center, Saudi Aramco, P.O. Box 5000, Dhahran 31311, Saudi Arabia
* Correspondence: ealsharaeh@alfaisal.edu

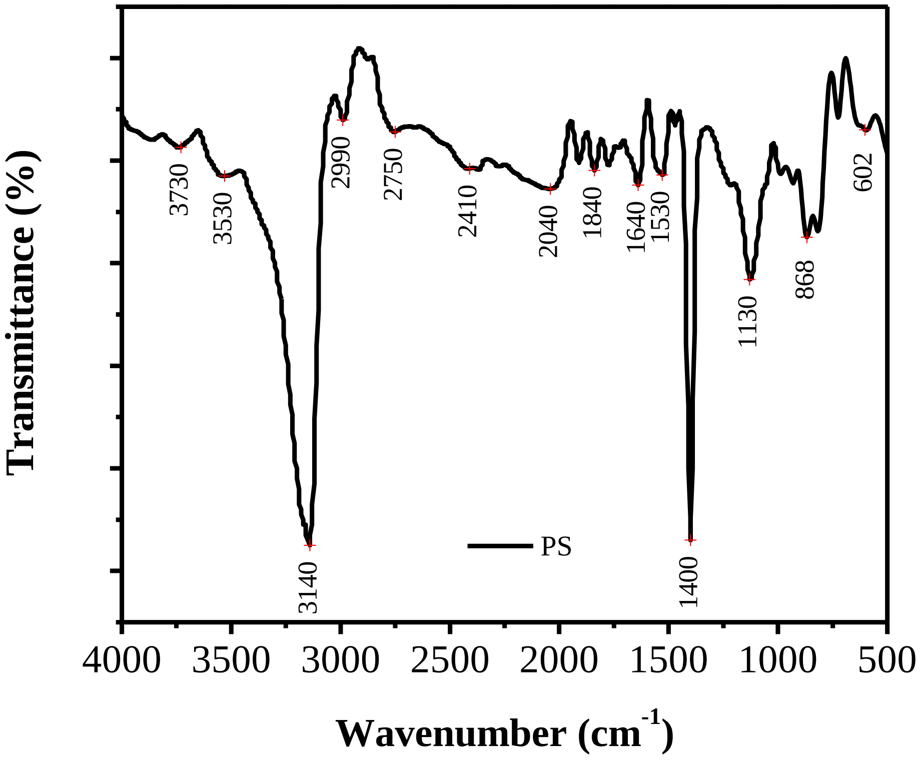


**Figure S1.** FTIR spectra of pure polystyrene.
